# Patient satisfaction with directly observed treatment and multidrug-resistant tuberculosis injection administration by lay health workers in rural Eswatini

**DOI:** 10.4102/phcfm.v12i1.2257

**Published:** 2020-05-26

**Authors:** Ernest Peresu, Christo J. Heunis, Gladys N. Kigoz, Diana De Grave

**Affiliations:** 1 Centre for Development Support, Faculty of Economic and Management Sciences, University of the Free State, Bloemfontein, South Africa; 2 Centre for Health Systems Research and Development, University of the Free State, Bloemfontein, South Africa; 3 Faculty of Business and Economics, University of Antwerp, Antwerp, Belgium

**Keywords:** community treatment supporter, human resources for health, task-shifting, multidrug-resistant tuberculosis, injection administration

## Abstract

**Background:**

The human resources for health crisis in rural Eswatini led to a novel community-based multidrug-resistant tuberculosis (MDR-TB) treatment strategy based on task-shifting, that is delegation of directly observed treatment (DOT) and administration of MDR-TB injections, traditionally restricted to professional nurses, to lay community treatment supporters (CTSs).

**Aim:**

This study assessed the level of patient satisfaction with receiving community-based MDR-TB care from a CTS.

**Setting:**

The study was conducted at three MDR-TB-treating facilities in the mostly rural Shiselweni region.

**Methods:**

A cross-sectional survey of a purposive sample of 78 patients receiving DOT and intramuscular MDR-TB injections from CTSs was carried out in 2017. Descriptive statistics and regressions were calculated.

**Results:**

A high overall general patient satisfaction score for receiving community-based MDR-TB care from a CTS was observed. Adherence counselling, confidentiality, provider selection and treatment costs significantly (*p* < 0.05) influenced satisfaction. A large majority (*n* = 62; 79.5%) of patients indicated that they would likely recommend their significant others to receive MDR-TB care from a CTS. Respondents identified the need to provide CTSs with adequate training, regular supervision and sufficient incentives and also to broaden the scope of their services.

**Conclusion:**

This study observed that task-shifting of DOT and MDR-TB injection administration to CTSs was supported from a patient perspective. However, adherence counselling, confidentiality, provider selection and treatment costs should be taken into account in community-based MDR-TB care programming. Further to the patients, community-based tuberculosis care could be enhanced by improving CTSs’ training, supervision and incentives, and broadening the scope of their services.

## Introduction

Tuberculosis (TB) is a major public health concern and remains one of the most devastating diseases in the world. Although global TB incidence has been declining for the past few years, a new threat has emerged – multidrug resistant-TB (MDR-TB) – that is showing no signs of receding and is undermining progress in TB control. MDR-TB is defined as strains of TB not susceptible to two of the most potent first-line drugs, isoniazid and rifampicin.^1^ Interrupted treatment of drug-sensitive TB is an important factor in the development of acquired drug resistance.^2^ In 2017, the World Health Organization (WHO) reported the proportion of MDR-TB in new and previously treated cases of TB in Eswatini to be 6.8% and 16.0% respectively.^1^ The country’s treatment success rate amongst MDR-TB cases remained at 68% (2015 cohort) and below the 2015 WHO target of 75% or higher.^3,4,5^ So serious is the problem of TB in Eswatini that the government declared the epidemic already in 2011 a national disaster.

In response, the Eswatini Ministry of Health developed the National TB Strategic Plan (2015–2019)^6^ that is aligned within the framework of the End TB Strategy^7^ and the Sustainable Development Goals (SDGs).^8^ The plan aims to strengthen community drug-resistant TB management, increase access to treatment and ensure that 75% or more MDR-TB cases are treated successfully.^6^

In Eswatini, the standardised therapeutic regimen for MDR-TB consists of toxic, complex and expensive second-line anti-TB drugs that include a daily injectable for the first 8 months and treatment that takes up to 24 months to complete.^9^ The National TB Control Programme (NTCP) is in the process of scaling up the use of the WHO-recommended shorter MDR-TB treatment regimens (9 months instead of the usual 2 years) across the whole country. Patients with MDR-TB typically make daily visits to the closest primary health care (PHC) facility to receive directly observed treatment (DOT) and their injection from a professional nurse. DOT is a strategy recommended by the WHO that involves a health worker supervising patients with TB as they take each dose of their standardised medication.^10^

In Eswatini, rural areas carry a disproportionately high burden of MDR-TB. In addition, PHC facilities are often distant and geographically inaccessible from patients’ homes and are also frequently characterised by lack of front-line TB human resources for health (HRH).^11,12,13,14^ In this context, front-line TB HRH refers to healthcare workers who are generally the community’s first level of contact within a PHC system for accessing TB services. To address the prevailing MDR-TB treatment access and HRH challenges in the predominantly rural Shiselweni region, in 2008, Médecins Sans Frontières (MSF) established a novel patient-centred community-based MDR-TB treatment strategy based on task-shifting within the existing NTCP. In this context, task-shifting refers primarily to the transfer of DOT and MDR-TB injection administration responsibilities, traditionally restricted to professional nurses, to trained lay community members. In extant literature, the task-shifting of highly differentiated clinical tasks such as injection administration to lay health workers has been limited to the delivery of contraceptives and injectable antibiotics in home-based management of newborn infections in rural and low-resource settings.^15,16^

Under this model of care, patients with MDR-TB discharged from the MDR-TB treating hospital are linked to a lay community member (not a family member) of their choice from their neighbourhood, referred to as a community treatment supporter (CTS). This strategy allows patients with MDR-TB to receive DOT and their daily injections from CTSs in their (the patients’) homes without having to make daily trips to the nearest clinic. The selected neighbour must have sufficient literacy skills essential for reading the training manual in English and documenting administered injections. On inception, newly recruited CTSs receive hands-on demonstrations, mentored practice and theoretical training that focuses primarily on MDR-TB epidemiology, transmission, diagnosis, treatment or strategies (DOT), safe injection handling and adverse drug reactions. In addition, because of the potential risk of contracting human immunodeficiency virus (HIV) infection from needle stick injuries, newly recruited CTSs are routinely informed of the patient’s HIV status prior to undergoing training. The CTS’s responsibilities include patient confidentiality and procedures for patient referral to community MDR-TB nurses and other MDR-TB team members.

CTSs are directly supported by a team of professional healthcare workers made up of a doctor, community MDR-TB nurse, psychologist and social worker. Each CTS receives on-going supervisory visits from and at the discretion of the community MDR-TB nurse, to re-evaluate their skills and on-the-job training needs. CTSs accompany their patients with MDR-TB to the main health facilities for their monthly outpatient treatment review or unscheduled visits, in the case of worsening health condition. Although CTSs are typically recruited and trained to focus solely on MDR-TB, they are also important adjuncts in the provision of adherence support to HIV co-infected patients with MDR-TB on antiretroviral therapy. Core clinical decisions related to the MDR-TB treatment are taken by formal facility-based professional healthcare workers. Local administration of DOT and intra-muscular injections by a CTS was introduced to reduce some of the social and financial difficulties frequently faced by patients in accessing the often more distant centralised clinic-based MDR-TB treatment services.^14^

Despite the decade-long implementation of the task-shifting strategy in the Shiselweni region, the Ministry of Health is yet to officially endorse the strategy. Existing NTCP strategic plans and MDR-TB treatment guidelines only provide for task-shifting of certain professional nurses’ responsibilities such as DOT supervision and TB treatment defaulter tracing to lay health workers. Professional bodies in Eswatini have voiced ethical concerns about standards of care and safety risks for both patients and CTSs ranging from potential errors in dosing to transmission of infections through unsafe injection handling and inappropriate community MDR-TB infection prevention and control practices.^17^ These fears were heightened by the increased recognition of the role community transmission played in the publicised outbreak of extensively drug-resistant TB in the neighbouring KwaZulu-Natal province of South Africa.^18^

Authorities have also raised questions regarding the sensitive nature of aspects of care delegated to lay community members, in particular, whether the CTSs respect patients’ confidential medical information.^17^ This is important given the stigma that surrounds the disease. Despite many years of public health education, MDR-TB is presumed to be incurable.^19,20^ CTSs learn about the medical condition and may view the intimate anatomy of patients as they administer and coordinate care. The CTS–patient encounter also takes place in the home setting, which is often not conducive for maintaining privacy and confidentiality.

Assessment of community-based MDR-TB services, specifically the task-shifting of care to CTSs, has often been restricted to clinical outcomes without accounting for patients’ satisfaction with health services received. There is strong evidence that patient satisfaction with healthcare services received can instrumentally affect treatment uptake, adherence and retention in TB care.^21,22,23,24,25^ The need to incorporate the patients’ perspective of TB care received is emphasised in the International Standards for Tuberculosis Care (ISTC)^25^ and the Compendium of Best Practice Guidelines of the International Union Against Tuberculosis and Lung Disease (The Union).^26^ Drawing from a myriad of existing WHO recommendations, the ISTC and The Union support the call for a patient-centred approach based on the patient’s needs and mutual respect between the patient and the healthcare service provider. By administering DOT and MDR-TB injections, CTSs perform a critical public health responsibility that carries a high level of obligation to the community and the individual MDR-TB patient. Thus, as frontline HRH in the provision of MDR-TB care, the interaction of CTSs with patients may considerably influence treatment outcomes.

Patient satisfaction with TB care refers to the match between the patient’s prior expectations of care and subsequent perception of services actually received.^19,20^ Patient satisfaction may be seen to centre on the fulfilment of the following expectations: reasonable waiting time for care, respectful and considerate interaction with healthcare providers, accurate diagnosis and optimism of a good prognosis, continuity and coordination of care, affordability and the professional competence of service providers.^21,22,27,28^

Despite the recognition of the importance of determining levels of patient satisfaction as a measure of the quality of TB services,^21,22^ no study to date has evaluated patients’ satisfaction with receiving DOT and intramuscular MDR-TB injections from CTSs in the Shiselweni region. In view of the growing global experience of the potential role of patient satisfaction in optimising clinical outcomes, particularly with respect to TB services, this study was conducted to assess the level of patients’ satisfaction and their perceptions of the different dimensions of MDR-TB care delegated from professional nurses to CTSs in a rural region of Eswatini. Results will help in formulating recommendations to optimise the task-shifting approach in community-based MDR-TB management in resource-limited settings and contribute to the achievement of various MDR-TB control targets as outlined in the national TB Strategic Plan, End TB Strategy and SDGs.

## Methods

### Study setting and design

The predominantly rural Shiselweni region is one of the four regions of Eswatini. With an estimated population of 204 111 in 2017,^29^ the region has one hospital and two health centres supporting 18 smaller outlying PHC clinics. In May 2017, a cross-sectional study was conducted amongst patients with MDR-TB receiving or who had received DOT and intra-muscular MDR-TB injections from a CTS in the Shiselweni region using a structured interviewer-administered questionnaire.

### Sampling method

The study population consisted of 124 patients with MDR-TB enrolled at any of the three MDR-TB-treating healthcare facilities in the Shiselweni region and receiving or who had received DOT and intramuscular injections from a CTS in the 12 months (to reduce recall bias) preceding May 2017. A purposive sample of 78 out of the study population was selected for the survey. A total of 46 patients with MDR-TB from the study population who did not meet the inclusion criteria were excluded from participation. The study excluded 26 patients younger than 18 years and seven participants who were too ill and hospitalised at the time of the study from participation.

Because of practical constraints, this study also excluded the participation of two patients with MDR-TB who had profound deafness from medication toxicity who could have provided diverse and instructive opinions about their level of satisfaction with the task-shifting community-based MDR-TB care to CTSs. The difficulty in comprehension could have undermined the potential deaf participants’ ability to give an informed consent, participate voluntarily and interact with researchers in the study. In addition, 11 patients with MDR-TB who had been under the care of a CTS for less than a month were not included as they may not have had enough experience to rate the services received from a CTS. There were no refusals to participate in the survey.

### Instrument development and data gathering

Given the paucity of an adequately tested appropriate tool to measure MDR-TB patient satisfaction with receiving DOT and intramuscular injections from CTSs, a newly developed questionnaire based on literature review, CTS training materials and job descriptions was used.^21,22,25,30^ The content validity of the questionnaire was assessed by expert opinion. The internal reliability of the satisfaction scales was also examined, considering Cronbach’s alpha coefficients of 0.7 or higher as acceptable.

The questionnaire consisted of items measuring socio-demographic characteristics of patients with MDR-TB including sex, age, marital status, educational level, employment status, source of income, duration of MDR-TB treatment, distance between place of residence and nearest PHC facility and types of transportation to the facility. Patient satisfaction with nine dimensions of MDR-TB care delegated to CTSs was measured by using single five-point Likert-scale items with responses ranging from ‘strongly disagree’ to ‘strongly agree’ and coded in values from 1 to 5. Both negatively and positively worded questions were included to minimise response bias. The questionnaire was forward-translated from English to siSwati and back-translated to English before being pre-tested for face validity and understanding in a convenience sample of 10 previous patients with MDR-TB who were excluded in the main study. The information from the pre-testing was used to revise and improve the clarity of the questions.

Trained interviewers conducted face-to-face interviews in either siSwati or English with patients attending one of the three rural health centres in the Shiselweni region. Patients with MDR-TB visiting these facilities for their monthly review in May 2017 were informed by the community MDR-TB nurse about the research at the end of their consultation. Participation in the study was voluntary and patients were assured that non-participation would not compromise their MDR-TB care in any way. No incentives or tokens of appreciation were provided. To reduce the effect of social desirability bias associated with self-reported satisfaction, participants were assured of the maintenance of confidentiality of the information gathered during the interviews. They were informed that no names or other personal identifying information would be collected during the interviews and that the findings would be reported anonymously using aggregate analysis. They were also assured that the outcomes of the study were for research purposes only and would not affect their care and support in any way.

Once a respondent agreed to participate, the trained interviewers explained the purpose of the research, data-gathering procedures, risks and benefits and the expected duration of the interview. Written informed consent was obtained from all study participants before the interviews were conducted.

### Measures

#### Satisfaction with dimensions of multidrug-resistant tuberculosis services from community treatment supporters

The questionnaire was divided into nine subscales covering satisfaction with dimensions of MDR-TB care and services received from CTSs. The breadth of measurement covered the following domains: (1) satisfaction with MDR-TB services availability and convenience (three items); (2) provider–patient communication (four items); (3) provider attitude, respect and compassion (five items); (4) stigma (one item); (5) health education (five items); (6) adherence counselling (one item); (7) confidentiality (one item); (8) provider selection (one item); and (9) treatment cost (one item).

#### General patient satisfaction

One global rating question regarding the primary outcome, general satisfaction with receiving DOT and intramuscular MDR-TB injections from a CTS was included. Another question enquiring about willingness to recommend a close family member or friend with MDR-TB to receive DOT and intramuscular MDR-TB injections from a CTS was also added as a proxy for general satisfaction with services provided by CTSs. To aid in the interpretation of the quantitative findings, an open-ended question ‘What can be done to improve community-based MDR-TB care services rendered by the CTS?’ was also included.

### Data analysis

Data from the questionnaire was double entered into Epi Info 7 and exported to Stata (Version 13.1) for analysis. Standard descriptive statistics were used to summarise socio-demographic variables. Mean scores were calculated for each dimension of MDR-TB care as a composite measure of satisfaction by using each of the items under the subscale indicator. Also, the mean score for the general satisfaction question was calculated.

Regression analysis was used to assess and compare the effect of socio-demographic variables on the general patient satisfaction score. Ordinal regression models were used to predict the regression coefficients for the mean general satisfaction score for each of the nine dimensions of MDR-TB services provided by CTSs. *P*-values of less than 0.05 were considered to be statistically significant. Individual participant responses to the open-ended question ‘What can be done to improve the services rendered by the CTSs?’ were reviewed, coded and manually assigned to key themes. By using thematic analysis, common ideas and emerging patterns in the responses from the data were identified and analysed.

### Ethical consideration

Ethical approval was obtained from the Scientific and Ethics Committee of Eswatini and the Health Sciences Research Ethics Committee (IRB00006240), University of the Free State. The NTCP and MSF authorised the study to be conducted. All eligible participants were provided with information about the study. Written consent was obtained from all participants (patients with MDR-TB) prior to their participation in the interviews.

## Results

Half of the participants (*n* = 39; 50.0%) were aged between 31 and 40 years (Table 1). Almost 9 in every 10 of the participants (*n* = 69; 88.5%) had received DOT and MDR-TB injections from a CTS for at least 4 months. Almost two-thirds (*n* = 47; 65.3%) were not married and more than half (*n* = 43; 55.1%) had low literacy and numeracy skills (primary education or lower). Most of the patients with MDR-TB interviewed (*n* = 65; 83.3%) were not employed. More than three-quarters of patients interviewed (*n* = 67; 85.9%) had an estimated monthly household income of less than ZAR1000 (= US$74.07 [exchange rate on 31 May 2017]). Most patients (*n* = 42; 53.8%) lived more than 20 km from the nearest PHC facility. A large majority (*n* = 75; 93.6%) relied on public transport to travel to the nearest healthcare facility.

**TABLE 1:** Socio-demographic characteristics of survey respondents.^31^

Characteristics	*N*	%
**Sex**		
Male	43	55.1
Female	35	44.9
**Age group (years)**		
< 30	20	25.6
30–39	39	50.0
40–49	7	9.0
≥ 50	12	15.4
**Marital status**		
Married	27	34.6
Unmarried	51	65.4
**Education level**		
Primary school or lower	43	55.1
Secondary school or higher	35	44.9
**Employment**		
Employed	13	16.7
Unemployed	65	83.3
**Estimated monthly household income (ZAR)†**		
0–1000	67	85.9
1001–10 000	11	14.1
> 10 000	0	0.0
**Duration on MDR-TB injections from CTS**		
1–4 months	9	11.5
> 4 months	69	88.5
**Distance to nearest clinic**		
< 5 km	5	6.4
5 km – 10 km	10	12.8
11 km – 20 km	21	26.9
> 20 km	42	53.8
**Transportation to clinic**		
Bus or taxi	73	93.6
Drive or ride from family member	3	3.8
Walk or bicycle	2	2.6

*Source:* Nabbuye-Sekandi J, Makumbi FE, Kasangaki A, et al. Patient satisfaction with services in outpatient clinics at Mulago hospital, Uganda. Int J Qual Health Care. 2011;23(5):516–523. https://doi.org/10.1093/intqhc/mzr040

MDR-TB, multidrug-resistant tuberculosis; CTS, community treatment supporter.

† , ZAR, South African Rand (local currency); US$1 = ZAR14.91 (exchange rate on 23 May 2017).

*N* = 78.

The overall mean score of general patient satisfaction for receiving DOT and intramuscular MDR-TB injections from a CTS was 4.4 (standard deviation: 1.0). Table 2 illustrates patient socio-demographic factors influencing mean general patients’ satisfaction scores. All socio-demographic factors were considered as predictor variables in the adjusted model. The study revealed no significant association between mean general satisfaction score and the other patient socio-demographic characteristics.

**TABLE 2:** Adjusted coefficients for mean general satisfaction and patient socio-demographic characteristics.

Characteristics	*N* (78)	Mean (4.4)	s.d. (1.0)	*p*	Adjusted			*p*
					*β*	95%	CI	
**Respondents’ sex**								
Male (reference)	43	4.4	1.0	0.8053	-	-	-	-
Female	35	4.4	1.0	-	0.12	−0.83	1.08	0.804
**Age group (years)**								
< 30 (reference)	20	4.2	1.2	0.2403	-	-	-	-
30–39	39	4.4	1.0	-	0.38	−0.71	1.48	0.493
40–49	7	4.3	1.1	-	0.05	−1.65	1.67	0.995
≥ 50	12	4.9	0.3	-	2.04	−0.18	4.26	0.072
**Marital status**								
Married (reference)	27	4.6	0.9	0.2849	-	-	-	-
Unmarried	51	4.4	1.1	-	0.58	−0.48	1.64	0.282
**Education level**								
Primary school or lower (reference)	43	4.6	0.8	0.378	-	-	-	-
Secondary school or higher	35	4.3	1.2	-	−0.43	−1.38	0.52	0.374
**Employment**								
Employed	13	4.2	1.2	0.7422	-	-	-	-
Unemployed	65	4.5	1.0	-	−0.23	−1.51	1.06	0.728
**Estimated monthly household income (ZAR)†**								
0–1000 (reference)	67	4.4	1.0	0.9579	-	-	-	-
1001–10 000	11	4.4	1.1	-	0.04	−1.37	1.45	0.956
**Duration on MDR-TB injections from CTS**				-	-	-	-	-
1–4 months (reference)	9	4.7	0.7	0.51	-	-	-	-
> 4 months	69	4.4	1.0	-	−0.54	−2.17	1.09	0.516
**Distance to nearest clinic**								
< 5 km (reference)	5	4.4	0.9	-	-	-	-	-
5 km – 10 km	10	4.3	1.1	0.91	−0.07	−2.14	2.00	0.945
11 km – 20 km	21	4.5	1.0	-	0.38	−1.54	2.30	0.699
> 20 km	42	4.4	1.1	-	0.35	−1.46	2.15	0.707
**Transportation to clinic**								
Bus or taxi	73	4.5	1.0	-	-	-	-	-
Drive or ride from family member (reference)	3	2.5	1.5	0.04	−3.58	−6.47	−0.69	0.015
Walk or bicycle	2	4.5	1.0	-	2.72	0.37	5.08	0.023

*Source:* Nabbuye-Sekandi J, Makumbi FE, Kasangaki A, et al. Patient satisfaction with services in outpatient clinics at Mulago hospital, Uganda. Int J Qual Health Care. 2011;23(5):516–523. https://doi.org/10.1093/intqhc/mzr040

MDR-TB, multidrug-resistant tuberculosis; CTS, community treatment supporter; 95% CI, 95% confidence interval; s.d., standard deviation.

† , ZAR, South African Rand (local currency); US$1 = ZAR14.91 (exchange rate on 23 May 2017).

A Spearman correlation coefficient was computed to examine the relationship between mean scores of general satisfaction and satisfaction with nine specific measures of MDR-TB services provided by CTSs (Table 3). There was a weak correlation between treatment costs, *r* (78) = 0.27, *p* = 0.015 and general satisfaction. Overall, the weak but positive correlation between treatment costs and general satisfaction scores suggests that this dimension assesses factors other than the patient’s general satisfaction.

**TABLE 3:** Correlation between mean general satisfaction score and satisfaction with other dimensions of multidrug-resistant tuberculosis care.

Satisfaction scale	Mean score		Correlation coefficients (*r*)	*P* < 0.05	*R*^2^
	Mean	s.d.			
Accessibility and convenience	Category 2	0.74	0.05	0.649	0.04
Provider–patient communication	4.87	0.37	0.10	0.392	0.09
Provider attitude, respect and compassion	4.77	0.52	0.01	0.947	0.09
Health education	4.78	0.78	0.14	0.214	0.29
Adherence counselling	4.91	0.40	−0.02	0.864	0.06
Stigma	4.41	1.16	−0.01	0.909	0.01
Confidentiality	4.78	0.49	−0.10	0.376	0.12
Provider selection	4.82	0.58	0.17	0.134	0.04
Treatment costs	4.65	1.03	0.27	**0.015**	0.28

*Source:* Nabbuye-Sekandi J, Makumbi FE, Kasangaki A, et al. Patient satisfaction with services in outpatient clinics at Mulago hospital, Uganda. Int J Qual Health Care. 2011;23(5):516–523. https://doi.org/10.1093/intqhc/mzr040

s.d., standard deviation.

All bold values are significant at *P* < 0.05.

Ordinal regression models were used to predict regression coefficients for the mean general satisfaction score for each of the nine dimensions of MDR-TB services provided by CTSs (Table 4). All nine dimensions of MDR-TB services provided by CTSs were included as potential explanatory variables in the adjusted model. In the adjusted model, adherence counselling (*p* = 0.019), confidentiality (*p* = 0.026), provider selection (*p* = 0.037) and treatment costs (*p* = 0.014) significantly predicted mean general patient satisfaction.

**TABLE 4:** Adjusted and unadjusted ordinal regression coefficients for mean scores of patient’s general satisfaction score by specific dimensions of multidrug-resistant tuberculosis care.

Dimensions of MDR-TB care scores	Unadjusted		Adjusted		*p*
	*β*	SE	*β*	SE	
Accessibility and convenience	0.42	0.30	−1.14	0.72	0.116
Provider–patient communication	1.37	0.60	−0.81	1.69	0.633
Provider attitude, respect and compassion	1.06	0.47	1.67	1.25	0.178
Health education	0.61	0.32	0.56	0.36	0.126
Adherence counselling	1.17	0.67	5.03	2.14	0.019
Stigma	0.04	0.20	−0.04	0.35	0.912
Confidentiality	0.21	0.62	4.58	2.06	0.026
Provider selection	0.56	0.32	1.73	0.83	0.037
Treatment costs	0.41	0.18	0.60	0.24	0.014

*Source:* Nabbuye-Sekandi J, Makumbi FE, Kasangaki A, et al. Patient satisfaction with services in outpatient clinics at Mulago hospital, Uganda. Int J Qual Health Care. 2011;23(5):516–523. https://doi.org/10.1093/intqhc/mzr040

MDR-TB, multidrug-resistant tuberculosis; SE, standard error.

Approximately four-fifths (*n* = 62; 79.5%) of the respondents indicated that they were likely to recommend their friends or family to receive DOT and MDR-TB injections from a CTS. More than half (*n* = 44; 56.4%) of the respondents suggested broadening of the scope of services provided by CTSs to include treatment of minor ailments or side effects of the medication such as nausea, vomiting and loss of appetite. More than one-third (*n* = 27; 34.6%) of the respondents identified the need to adequately train CTSs on respecting the privacy and confidentiality of their condition and medical information. Just more than a quarter (*n* = 20; 25.6%) of patients highlighted the need for regular supervision of and sufficient incentives for CTSs as ways of improving MDR-TB services.

## Discussion

Previous studies evaluating community-based MDR-TB models of care have focused on clinical outcomes^32,33^ without paying attention to patients’ satisfaction. As far as could be ascertained, this is the first study to assess MDR-TB patients’ views and the extent to which their perceptions of various aspects of care experiences provided by CTSs are associated with overall satisfaction with community-based MDR-TB treatment and care in the rural Shiselweni region. This study further contributes to the current national debate on task-shifting of DOT and injection administration to lay community members by providing the patients’ perspective and suggestions of ways to improve patient experiences in practice.

The overall mean score of self-reported general patient satisfaction in this study was fairly favourable: 4.4 based on a rating scale ranging from 1 (very low satisfaction) to 5 (very high satisfaction). There is a paucity of literature describing patients’ perspectives on receiving MDR-TB treatment, with most researchers limiting their assessment to patient satisfaction with drug-sensitive TB services. Patient satisfaction is considered to be a highly desirable outcome measure of clinical care, influencing both treatment adherence and continuity of care. Findings from studies on levels of general satisfaction with community-based TB services conducted in various African settings in diverse geographical locations, albeit limited in number, have often been inconsistent.^21,22,24,30^ The mixed results are not unexpected because there are huge variations in how TB care is offered across different settings.

In studies conducted in South Africa^22^ and Nigeria,^24^ patients’ socio-demographic characteristics such as age, gender and marital status have been reported to contribute significantly to patient satisfaction. Conversely, in this study patient characteristics were not significantly related to patient satisfaction, with the exception of the mode of transport. Patients with MDR-TB in this study also suggested the need to broaden the scope of services provided by CTSs to include treatment of minor ailments and side effects of the medication. Taken together, these findings provide evidence that patients in poor rural settings face many barriers in accessing PHC such as transport limitations in reaching the distant and geographically inaccessible clinics.^12,14^ This is significant in the light of findings from a study in rural South Africa that reported time lost travelling for TB treatment as a potential cause of missed appointments and threat to treatment adherence and retention.^34^ The findings from this study provide an impetus for policymakers to consider progressively increasing, where applicable, the geographical distribution of PHC facilities in rural areas. There is also a need for more research to understand the extent to which transport limitations impede access to MDR-TB services in rural settings.

In this study, four dimensions of care experience – adherence counselling, confidentiality, provider selection and treatment costs – were found to significantly predict overall patient satisfaction. These findings could assist authorities in framing specific strategic interventions to improve patient satisfaction and strengthen patient-centred community-based MDR-TB care. It was rather surprising to find that the other dimensions of MDR-TB care provided by CTSs were not related to general patient satisfaction because these aspects have been linked to satisfaction in prior studies.^21,22,23,24,27,30,35^

MDR-TB treatment regimens are lengthy with a high daily pill burden carrying frequent side effects and requiring high levels of adherence to function optimally. Yet, the level of adherence to MDR-TB treatment varies significantly in different settings.^36,37,38^ As such, adherence counselling is considered an integral component of a worthwhile care experience.^21,24^ Consistent with results from previous research,^24^ this study found adherence counselling to significantly influence patient satisfaction. These results suggest key targets to optimise the delivery of community-based MDR-TB care by CTSs. The WHO^39^ and the Eswatini treatment guidelines^40^ for drug-resistant TB recommend that healthcare workers should regularly assess, reinforce and support patient adherence to complex MDR-TB treatment plans because of its importance to successful treatment. Ongoing support for CTSs should recognise their critical role in adherence counselling and reinforce their training about the disease, treatment methods, common complications and skills essential to optimise adherence to care. On the basis of this finding, further research is needed to understand adherence counselling practices of CTSs that may guide the development of comprehensive MDR-TB treatment adherence interventions.

Findings from this study point to treatment costs as one of the dimensions of MDR-TB care that predicted general patient satisfaction. Although MDR-TB treatment is generally offered free of charge, a considerable share of the costs are incurred by patients and their households. Patients with MDR-TB are confronted with costs associated with visits to the healthcare facilities such as transport, food and accommodation and loss of income because of inability to work. These costs impose a financial strain on patients and their households, potentially creating barriers to treatment access and adherence and increased treatment interruptions.^41,42^ In the present study, a majority of patients were unemployed and reported a low monthly household income. One of the main milestones of the End TB strategy is that no families affected by TB face catastrophic costs by 2020.^6^ As such, various levels and types of social protection support measures should be considered by policymakers to assist patients with MDR-TB and their families. Future studies should assist policy makers in identifying, understanding and mitigating the effect of MDR-TB treatment on the financial status of patients and their families.

This study found that provider selection influenced general patient satisfaction. Perrault^43^ established that patients who purposely participated in choosing their healthcare provider reported higher satisfaction. These patients often preferred to choose providers with whom they felt they shared some similarity. Patients with MDR-TB in this study context purposely selected a CTS of their choice from their neighbourhood. Thus, the CTSs worked in the community they lived, typically sharing similar culture, language, religion, socio-economic status and life experiences with their patients with MDR-TB. The findings of the current study may suggest that provider selection empowered patients with MDR-TB to play a significant involvement in their own care by participating in arguably one of the most key decisions – the initial nomination of a DOT and injection provider. This is important given the recent calls by international bodies challenging national TB programmes to develop a broader interest in patient-centred care and systematically consider the perspectives of patients in the planning and provision of TB care.^25,26^

Prior to decentralisation of MDR-TB treatment, few people other than professional healthcare workers were de facto custodians of patients’ health information, a situation that has changed with the task-shifting of clinical tasks to CTSs in the Shiselweni region. The observation of the confidentiality of patients’ medical information is recognised as a central tenet in engendering patient satisfaction with TB care.^22^ The present study found a link between the overall satisfaction of patients with MDR-TB and their perception of the maintenance of confidentiality as they interacted with CTSs. A breach of confidentiality can erode patients’ trust in their healthcare provider and may have grave repercussions such as marital difficulties, discrimination and loss of employment.^19,20^ This observation is important in the light of an assertion made by Dapaah and Senah^44^ in a study carried out in rural Ghana on the socio-psychological trauma patients experience in accessing HIV services. The authors reported that patients were actually more worried about the social consequences of treatment than the medical outcomes of accessing care.

The task-shifting of DOT and intramuscular MDR-TB injections to lay healthcare workers in the Shiselweni region has so far gone ahead despite reservations and apprehensions from policymakers and NTCP functionaries.^17^ Policymakers have raised ethical concerns related to the perceived inability of lay health workers to observe the privacy and confidentiality of patients and also recognise and report the adverse events of MDR-TB treatment such as hearing loss. A substantial proportion of patients with MDR-TB in this study recommended the need for adequate training on respecting patients’ confidentiality, regular supervision and appropriate incentives for CTS. Implicitly, these suggestions potentially represent concerns from participants about CTSs’ deficiencies in those aspects of care. Therefore, it is recommended that authorities consider formalising the task-shifting practice and facilitate the development of appropriate competency-based pre-service and on-the-job training, optimisation of supportive supervision of CTSs and active surveillance of treatment adverse events and breaches of confidentiality in the community based MDR-TB treatment strategy. Future studies could examine ways to improve CTSs’ respect for and maintenance of the confidentiality of patients’ medical information.

In this study, almost four in every five of the respondents indicated that they were likely to recommend to their friends or family to receive community-based MDR-TB care from a CTS. This is significant in the context of scaling up the community-based programme implemented in the rural Shiselweni region. Without discounting the significance of other factors, the fact that patients with MDR-TB would recommend to their significant others to receive care from a CTS is considered a valuable indicator of their personal experience of receiving services from a community-based healthcare provider.^45,46^ The programme’s reputation (or level of former or current patients’ satisfaction) is likely to be the only information available to prospective patients with MDR-TB or their care givers to help them choose a preferred provider or model of care. Previous satisfaction is also important for former patients to return for further care to the same healthcare provider or programme in the future. The present study findings reveal new insights for programme managers and policy makers targeting new patients with MDR-TB, retaining existing ones and generally increasing access to MDR-TB care.

## Limitations

The use of patient perspectives to assess satisfaction with aspects of care by providers is not without problems. Tension exists regarding the subjective nature of patients’ evaluation and questions have been raised as to whether their views truly represent the quality of care or the healthcare workers’ interpersonal skills.^22^ Patient satisfaction is considered to reflect the service dimension rather than the technical aspect of quality of care.^22^ Participants’ responses may also reflect socially desirable answers rather than their true views and experiences. With this study mostly founded on self-reported satisfaction, social desirability bias may have occurred. However, this was partially countered by assuring respondents of the anonymity of the questionnaires that findings will be reported by using aggregate analysis and that the outcomes of the study would not affect their care in any way.

The sample for this study was relatively small (*n* = 78), and participants were selected purposively. Small samples may be inadequate to provide reliable correlations. Furthermore, the strength of association between general patient satisfaction and the dimensions of care that were significant was weak. Although the current study included a number of possible underlying dimensions of care influencing satisfaction, future studies with a broadened spectrum of dimensions of care may explain some of the differences in patient satisfaction reported in this research. In addition, satisfaction with healthcare services cannot be adequately assessed without accounting for a patient’s self-perceived health status. Literature suggests that the overall satisfaction rating is significantly influenced by the patient’s severity of illness.^47,48^ Although the present study excluded patients with MDR-TB who were too ill or had profound deafness from medication toxicity, future studies on patient satisfaction with the community-based MDR-TB model should include a measure of the participant’s health status.

In spite of these limitations, the findings from this study provide valuable insights on how the services provided by CTSs are perceived by patients and what authorities may have to prioritise to improve patients’ satisfaction.

## Conclusion

The findings provide strong evidence that patients were satisfied with the task-shifting of DOT and MDR-TB injection administration to CTSs in the study area. The study also provided some insights into the four dimensions of care grounded in adherence counselling, confidentiality, provider selection and treatment costs that significantly influenced patients’ general satisfaction. These aspects of MDR-TB care will need to be addressed to improve overall patient satisfaction. Provision of adequate training, regular supportive supervision, sufficient incentives and broadening of the scope of services offered by CTSs to include treatment of minor ailments were recommended as ways to optimise the delivery of community-based MDR-TB care.

The expansion of tasks delegated to lay health workers to include administration of important injection medication has great potential. Evidence from this study suggests the need for policy and regulatory support to formalise the task-shifting practice; developing interventions for active surveillance of treatment of adverse events; and improving patient experience of privacy and confidentiality. Future studies could further explore the barriers to and facilitators of the delivery of patient-centred MDR-TB care based on the task-shifting of clinical tasks to lay health workers.
